# Hierarchically 2D Porous Nanosheets Derived from Clay Minerals for Enhanced Carbon Dioxide Capture

**DOI:** 10.3390/nano16171089

**Published:** 2026-08-31

**Authors:** Changrui Shi, Wenyue Xue, Zhen Ma, Jing Hao, Guangsong Si, Hongwei Li, Wenguang Geng, Huiquan Liu

**Affiliations:** 1 Energy Research Institute, Shandong Engineering Research Center for Energy Efficiency and Low Carbon, Qilu University of Technology (Shandong Academy of Sciences), Jinan 250014, China; crshi@qlu.edu.cn (C.S.); wyxue6@163.com (W.X.); haojing202608@163.com (J.H.); 2 Energy Conservation and Dual Carbon Promotion Center of Shandong Province, Jinan 250011, China; mazhen310@163.com; 3 Shandong Precise Communications Technology Co., Ltd., Jinan 250100, China; siguangsong@pusaitongxin.com (G.S.); lihongwei@pusaitongxin.com (H.L.); 4 College of Power and Energy Engineering, Harbin Engineering University, Harbin 150001, China

**Keywords:** clay, nanosheets, vermiculite, gas adsorption, carbon dioxide capture

## Abstract

Carbon dioxide (CO_2_) capture and storage technologies are key methods for mitigating the greenhouse effect. The development of high-performance adsorbents using abundant natural minerals represents a promising approach to achieving cost-effective CO_2_ capture. In this work, we report a simple and scalable strategy for preparing two-dimensional (2D) porous nanosheets from the natural layered clay mineral vermiculite as the raw material, via liquid-phase exfoliation and acid etching. The clay-based 2D porous nanosheets exhibited a high specific surface area, a hierarchical porous structure, and mechanical and chemical stability. In the presence of the 2D porous nanosheets, the CO_2_ capture and adsorption capacity were significantly enhanced. The gas adsorption properties of the porous nanosheets were investigated over a wide temperature range (25–75 °C), achieving a gas uptake capacity of 38.22 mmol/g (at 75 °C and 35 bar). Furthermore, an intrinsic relationship between the pore structure of the porous nanosheets and their gas adsorption performance was revealed. The maximum uptake capacity remained at approximately 36.40 mmol/g during five adsorption–desorption cycles conducted at 75 °C. This work provides a promising approach for further development of clay-derived adsorbents for CO_2_ capture and may also offer useful implications for understanding CO_2_ geological sequestration.

## 1. Introduction

Rapid global industrialization has required the consumption of large quantities of fossil fuels, leading to a sharp rise in carbon dioxide (CO_2_) emissions [1,2]. Excessive CO_2_ emissions into the atmosphere are considered a major factor contributing to the greenhouse effect and triggering other harmful environmental impacts [3,4,5]. CO_2_ capture and storage (CCS) technology is regarded as a reliable method for reducing carbon emissions and tackling climate change [6,7].

CCS technology captures CO_2_ emitted by heavy industry and power generation. The captured CO_2_ is transported to storage sites (such as deep geological formations) for long-term storage, thereby significantly reducing carbon emissions [8,9]. The cost of the capture stage typically accounts for approximately 80% of the total cost of the entire CCS chain. Capture efficiency and energy consumption directly determine the economic viability and feasibility of CCS technology [10,11]. Developing energy-efficient and low-energy-consumption CO_2_ capture methods is a pivotal prerequisite for advancing CCS technology toward practical, large-scale application [12]. Compared with traditional chemical adsorption and low-temperature distillation, CO_2_ capture methods based on solid adsorbents have attracted widespread attention from researchers due to their non-corrosive nature and low regeneration temperatures [13,14,15].

Encouraging progress has been made in the design and performance optimization of solid adsorbents such as porous carbon materials [16], zeolites [17], and metal–organic frameworks [18]. Benefiting from their high specific surface area, readily accessible active sites, and short diffusion paths, two-dimensional (2D) porous materials have emerged as highly promising adsorbents for CO_2_ capture [19,20]. Over the past decade, substantial research has been conducted in the preparation of 2D porous materials, including graphene [21], layered double hydroxide [22], boron nitride [23], and MXene [24]. In-depth investigations have been conducted into the control of the pore structure and surface chemistry of 2D porous materials to achieve superior CO_2_ adsorption performance [25,26]. For the commercial application of CCS technology, solid adsorbents must offer not only excellent capture performance but their cost-effectiveness and simplicity of synthesis are equally crucial. Natural layered aluminosilicates (commonly known as clays) offer highly attractive advantages due to their abundant reserves, low cost, as well as mechanical and thermal stability. The exfoliation of layered clays into 2D nanosheets offers a promising avenue for the development of high-performance CO_2_ capture materials [14,27]. In addition, as layered clays are the primary diagenetic components in sedimentary strata, elucidating the structure–property relationships of clay-based CO_2_ adsorbents contributes to a deeper understanding of geological CO_2_ storage [28].

In this work, we used the natural clay mineral vermiculite as the raw material to prepare 2D porous nanosheets with a high specific surface area and a hierarchical porous structure, employing a simple strategy that is easily scalable. We investigated the CO_2_ adsorption behavior of the clay-based porous nanosheets over a wide temperature range (25–75 °C) and achieved a high CO_2_ uptake capacity of 56.09 mmol/g at 25 °C and 45 bar. Furthermore, the clay-based porous nanosheets demonstrated excellent cycle stability. After undergoing five adsorption–desorption cycles at 75 °C, the CO_2_ capture capacity remained at approximately 36.40 mmol/g. Our research findings provide insights for the rational design of highly efficient adsorbents, thereby advancing the application of industrial-scale flue gas carbon capture technology.

## 2. Materials and Methods

### 2.1. Materials

Natural vermiculite particles (VP, average sizes of 1–3 mm) were ordered from Hebei Lingshou Ore Powder Factory (Shijiazhuang, China). Sodium chloride (NaCl), lithium chloride (LiCl), and hydrochloric acid (HCl) were purchased from Sinopharm Chemical Reagent Co., Ltd. (Shanghai, China). CO_2_ gas (purity of 99.99%) was bought from Dalian Special Gases Co., Ltd. (Dalian, China). Deionized water (18.2 MΩ·cm) was prepared via reverse osmosis in our lab (EMOAS AD2L-10-BE, Shanghai, China).

### 2.2. Preparation of Clay-Based Porous Nanosheets

In the typical synthesis, a vermiculite nanosheet (VNS) suspension was prepared by ion intercalation-assisted liquid phase exfoliation, as we previously reported [29]. The VNS suspension was frozen at −18 °C for 2 h and subsequently freeze-dried at −43 °C for 48 h to obtain solid VNS. The as-made VNS was mixed with 0.1 M HCl at a solid-to-liquid ratio of 4 g per 2 L. After vigorous stirring for 12 h, the mixture was left to stand for another 12 h. The lower precipitate was collected, washed several times with deionized water by centrifugation, and subjected again to freezing and freeze-drying to yield acid-treated VNS (AT-VNS). For comparison, VP were mixed with HCl and stirred according to the same procedure, followed by washing, freezing and freeze-drying, to prepare acid-treated VP (AT-VP).

### 2.3. Characterization

The detailed morphologies of the samples were analyzed by scanning electron microscopy (SEM, SU8220) operated at 5.0 kV and 10.0 μA. The crystal structures and chemical compositions of the samples were measured by X-ray diffraction (XRD, Bruker D8 Advance, λ = 0.154 nm) with filtered Cu Kα radiation, Fourier transform infrared spectroscopy (FT-IR, Nicolet 6700) in the range of 4000–400 cm^−1^, and X-ray photoelectron spectroscopy (XPS, ESCALAB 250Xi) in the region from 1350 to 0 eV. The nitrogen sorption isotherms of the samples were collected using a surface area and porosity analyzer (Quadrasorb SI) at cryogenic temperatures (−196 °C).

### 2.4. CO_2_ Adsorption Measurements

CO_2_ adsorption measurements were performed using a high-pressure volumetric analyzer (HPVA II-200, Micromeritics). Prior to each measurement, the sample was degassed under vacuum at 80 °C for 12 h to remove physisorbed moisture and other volatile impurities. CO_2_ adsorption isotherms were recorded at three different temperatures (25, 50, and 75 °C) and within the pressure range of 0–45 bar. The adsorption equilibrium condition was defined as a pressure change of less than 0.03 bar/min. The temperature was controlled with a circulating bath (for 25 °C) or a heating mantle (for 75 °C). To evaluate the cyclic adsorption stability, five consecutive CO_2_ adsorption–desorption cycles were carried out at 75 °C.

## 3. Results and Discussions

### 3.1. Synthesis, Morphology, and Composition of Clay-Based Porous Nanosheets

**Figure 1:**
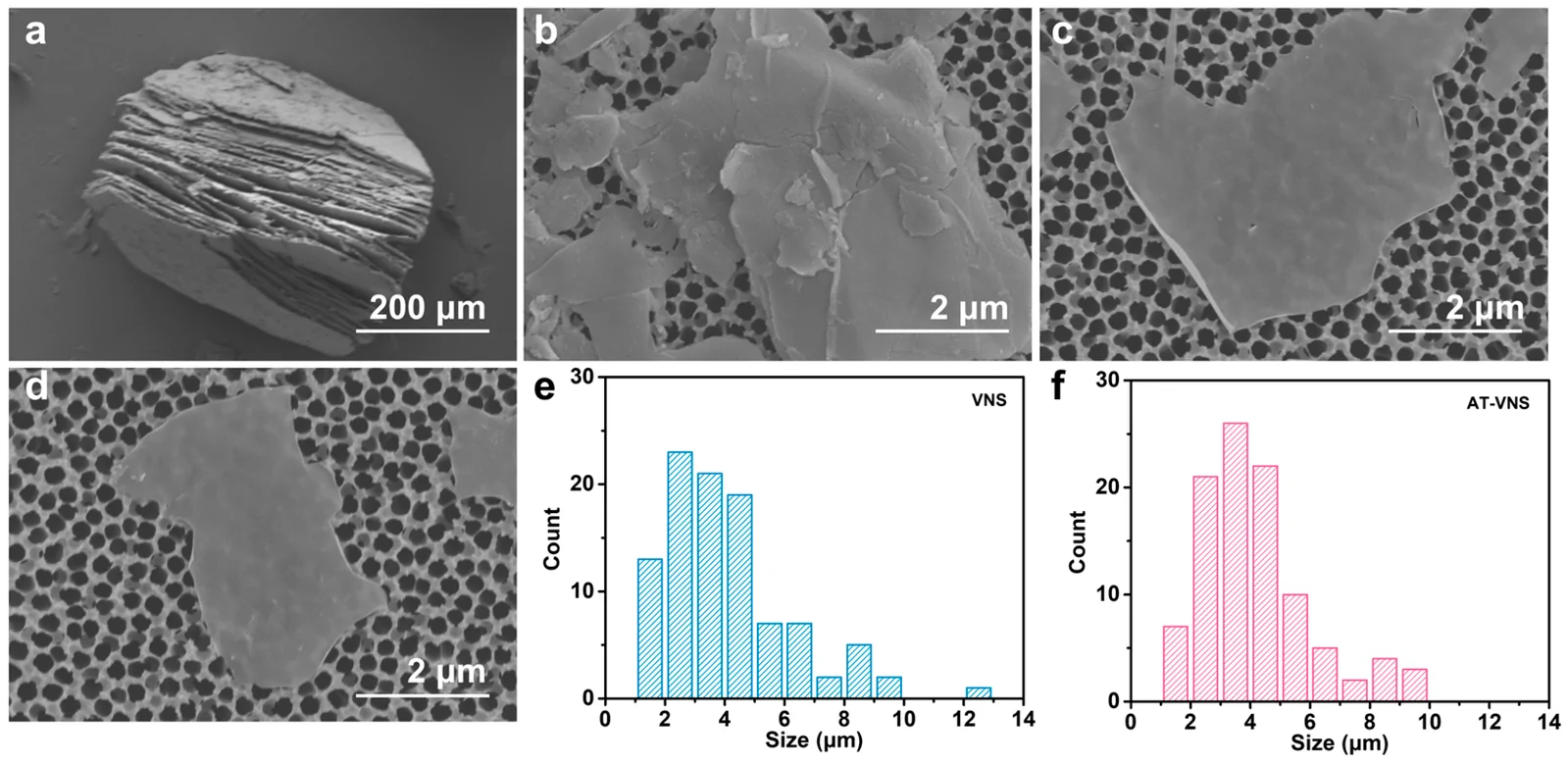
SEM images of (**a**) VP, (**b**) AT-VP, (**c**) VNS, and (**d**) AT-VNS. Lateral size distributions of (**e**) VNS and (**f**) AT-VNS (100 nanosheets analyzed).

Vermiculite is a typical clay mineral with a 2:1 layered silicate structure. Natural vermiculite particles have an accordion-like structure, comprising stacked crystal layers (Figure 1a), which enables vermiculite to be exfoliated into 2D nanosheets through chemical or physical processes. It can be observed that the AT-VP obtained by direct acid etching of VP takes the form of thick flakes (Figure 1b). The leaching effect of HCl on interlayer cations weakens the bonding forces between the layers, resulting in local delamination of the VP. The VNS prepared using a top-down exfoliation strategy exhibits a large lateral size and ultra-thin feature, as the porous substrate (anodized aluminum oxide membrane) under the nanosheet can be clearly identified (Figure 1c). The 2D nanosheets expose a large number of surface atoms, which serve as abundant adsorption sites and create favorable conditions for CO_2_ capture. As shown in Figure 1d, there is no significant difference in the microstructure between the acid-etched nanosheets and the unetched nanosheets. The statistical analysis of the lateral size distribution based on SEM images further confirms that the size distribution characteristics of the VNS and AT-VNS samples are very similar (Figure 1e,f). Therefore, the acid treatment did not result in lateral fragmentation, curling, or restacking of the nanosheets.

**Figure 2:**
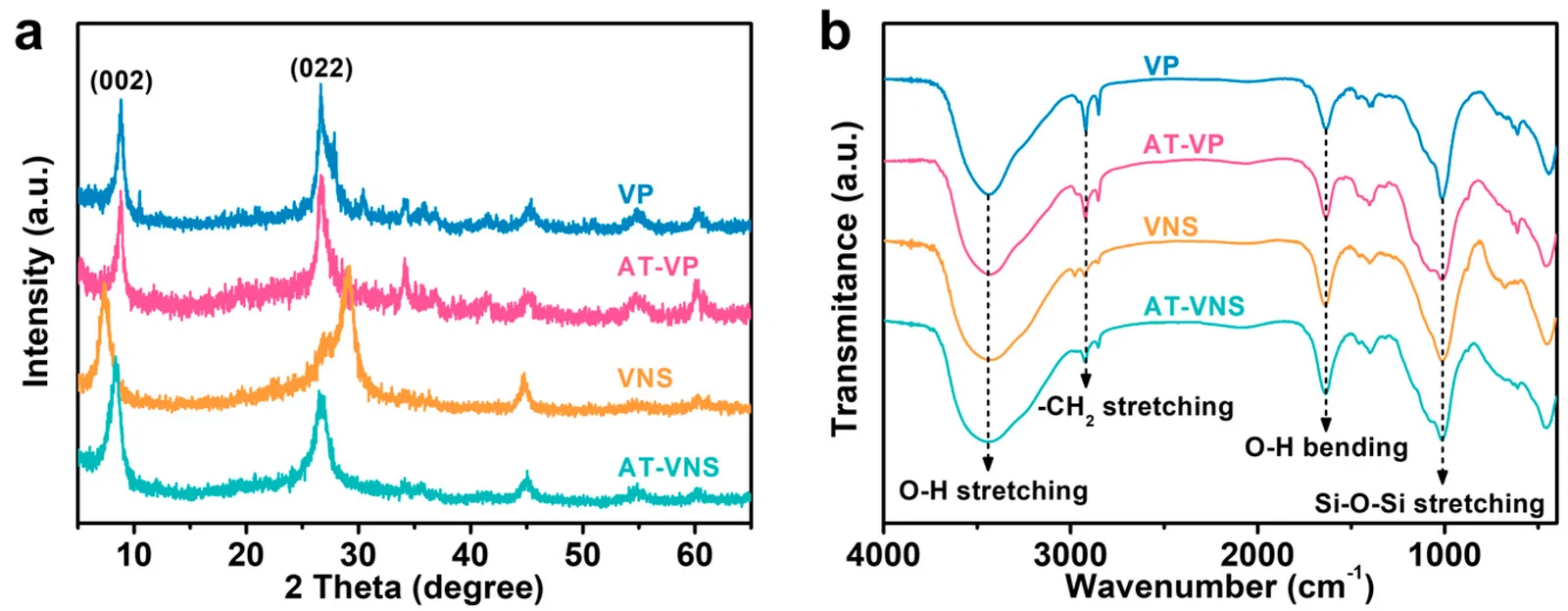
(**a**) XRD patterns and (**b**) FT-IR spectra of VP, AT-VP, VNS, and AT-VNS.

Figure 2a shows the XRD patterns of the raw particles and the clay-based porous nanosheets. All samples exhibited two diffraction peaks corresponding to the (002) and (022) crystallographic planes [30]. The diffraction peak of the (002) crystallographic plane for both VP and AT-VP was located at 8.8°, indicating that direct acid treatment of VP did not significantly disrupt the layered crystal framework. The shift of the (002) reflection from 8.8° to 7.3° provides evidence of structural expansion/exfoliation of vermiculite [31]. The (002) diffraction peak of AT-VNS was located at 8.3°, and the half-width at half-maximum remained relatively broad, indicating that the layered nanostructure was preserved following acid etching. The observed rightward shift of the (022) reflection for VNS compared to VP is attributed to lattice contraction induced by interlayer dehydration and mechanical exfoliation stress. As shown in Figure 2b, two bonds at 3443 and 2921 cm^−1^ in the FT-IR spectra correspond to O-H and -CH_2_ stretching vibration, respectively [32]. The enhancement of the O-H stretching vibration in the VNS and AT-VNS spectra was attributed to the increased exposure of hydroxyl active sites on the nanosheets. The characteristic bond at 1633 cm^−1^ originates from the stretching vibration of O-H. In addition, the strong band at 1014 cm^−1^ was assigned to the Si-O-Si stretching vibration [33].

**Figure 3:**
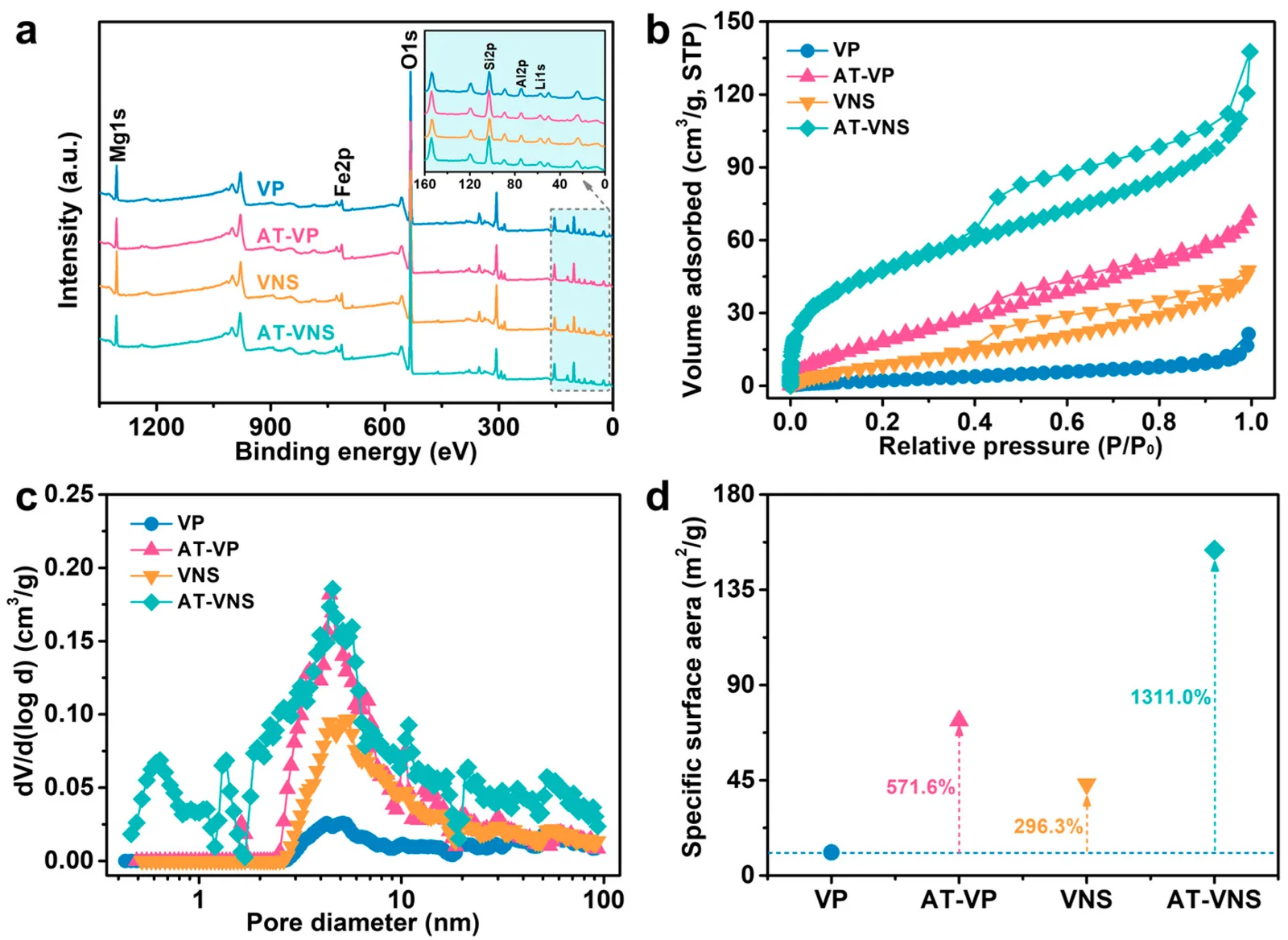
(**a**) XPS survey spectra, (**b**) nitrogen adsorption–desorption isotherms, (**c**) the pore size distributions, and (**d**) specific surface areas of VP, AT-VP, VNS, and AT-VNS.

XPS analysis was used to investigate the surface compositions of the raw particles and the clay-based porous nanosheets (Figure 3a). The Mg1s, Fe2p, O1s, Si2p, Al2p, and Li1s peaks contained in the XPS survey spectra can be observed. The ratios of Si to Al in the VP and VNS samples were 1.9 and 2.1, respectively, whilst those in the AT-VP and AT-VNS samples increased to 3.0 and 2.7. This phenomenon is attributed to the fact that the leaching rate of Al from tetrahedral and octahedral structures was significantly higher than that of Si during the acid treatment process. The pore size distributions and specific surface areas were investigated using nitrogen adsorption–desorption (Figure 3b–d). The nitrogen adsorption–desorption isotherms are type IV isotherms with a distinct H4 hysteresis loop (Figure 3b). The macroporous structure was confirmed by the adsorption isotherms in the high relative pressure region (P/P_0_ ≥ 0.9). The steep increase of AT-VNS in the low relative pressure region (P/P_0_ ≤ 0.01) indicates that it possesses a rich microporous structure [34]. The pore size distribution results indicate that the hierarchical porous structure of AT-VNS consists of micropores with diameters of 0.4–2.0 nm, mesopores with diameters of 3.0–20.0 nm, and macropores with diameters of 30.0–100.0 nm (Figure 3c). The specific surface areas of VP, AT-VP, VNS, and AT-VNS were 10.9, 73.2, 43.2, and 153.8 m^2^/g, respectively (Figure 3d). It is worth noting that the specific surface area of AT-VNS obtained via liquid-phase exfoliation and acid etching increased by 1311.0% compared with the raw particles.

### 3.2. CO_2_ Adsorption Performance of Clay-Based Porous Nanosheets

**Figure 4:**
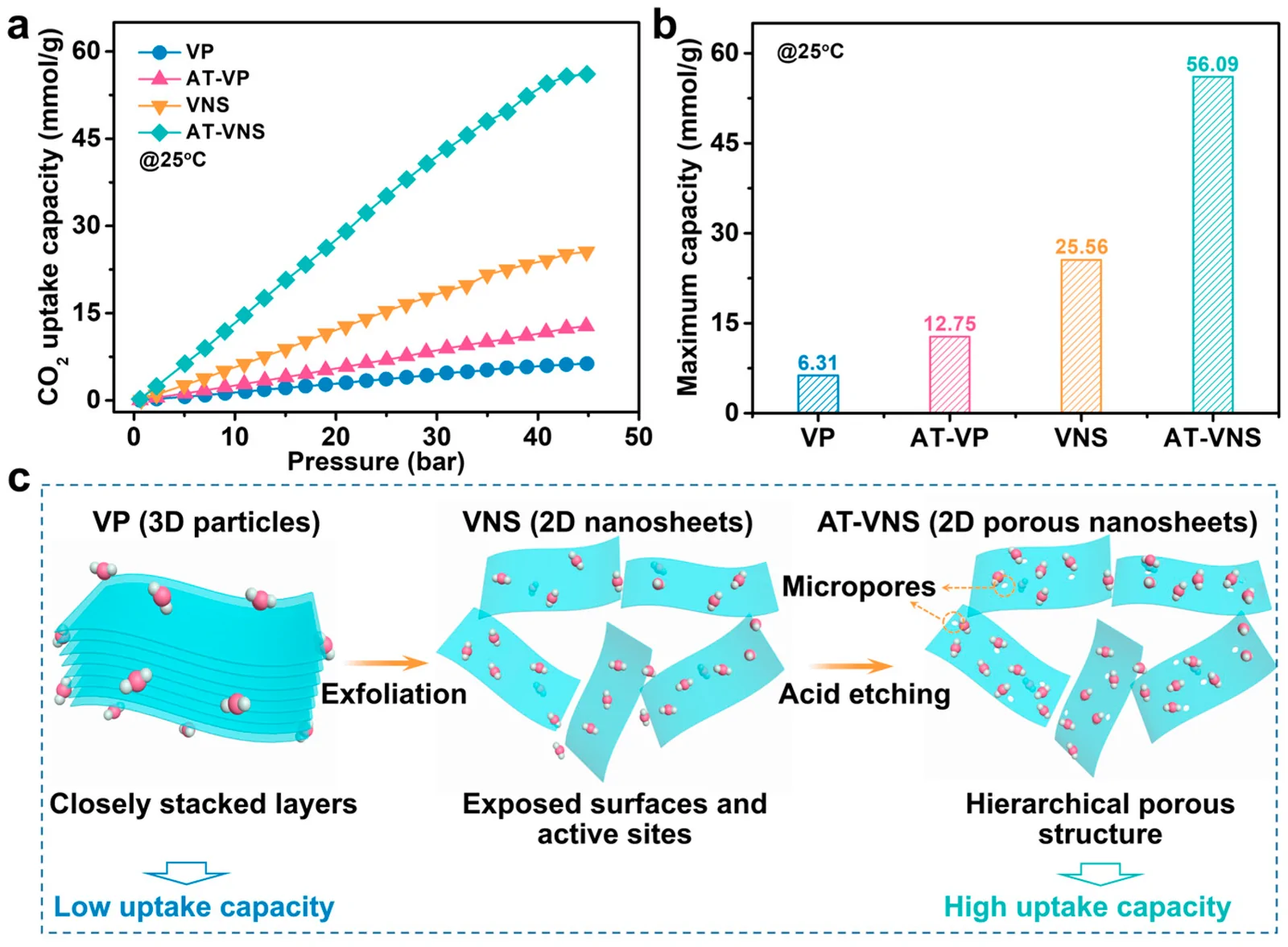
(**a**) CO_2_ adsorption isotherms and (**b**) maximum uptake capacity of VP, AT-VP, VNS, and AT-VNS at 25 °C. (**c**) Schematic illustration for CO_2_ adsorption on clay-based porous nanosheets.

The exposed surfaces, high specific surface area, and hierarchical porous structure of clay-based porous nanosheets provide excellent prerequisites for enhancing CO_2_ adsorption performance. The CO_2_ adsorption isotherms and maximum uptake capacity of the as-made samples at 25 °C are shown in Figure 4a,b. The CO_2_ adsorption capacity of all samples increased with rising pressure across the pressure range of 0–45 bar. The maximum uptake capacities of VP, AT-VP, VNS, and AT-VNS were 6.3, 12.75, 25.56, and 56.09 mmol/g, respectively (Figure 4b). AT-VNS exhibited the highest uptake capacity, which is comparable or even higher than many of the previously reported results (Table 1). Although VNS has a lower specific surface area than AT-VP, it exhibits a higher CO_2_ uptake capacity, which is attributed to the open-pore structure and increased hydroxyl active sites of VNS. Therefore, the CO_2_ uptake capacity of clay-based porous nanosheets is influenced by a combination of factors, including specific surface area, pore structure, and surface chemistry. The maximum uptake capacity of AT-VNS was 8.9 times higher than that of the raw material, demonstrating the synergistic effect of liquid-phase exfoliation and acid etching in enhancing CO_2_ capture performance.

Figure 4c schematically illustrates the mechanism of CO_2_ adsorption on clay-based porous nanosheets. The raw material VP consists of numerous closely stacked layers, the surfaces of most of which are enclosed within the particles. The three-dimensional (3D) particle structure provides a very limited surface area for CO_2_ adsorption. 2D nanosheets with exposed surfaces and active sites were prepared using a strategy involving ionic intercalation-assisted liquid phase exfoliation. Acid etching produces a microporous structure without compromising the structural integrity of the nanosheet layers, thereby yielding a hierarchical porous structure comprising micropores, mesopores, and macropores. For CO_2_ adsorption processes, it is proposed that the mesopores in hierarchical porous structures facilitate the free diffusion of gas molecules, while the micropores provide stable adsorption sites [15,40]. Therefore, the exposed surfaces and hierarchical porous structure confer a high uptake capacity on AT-VNS.

**Figure 5:**
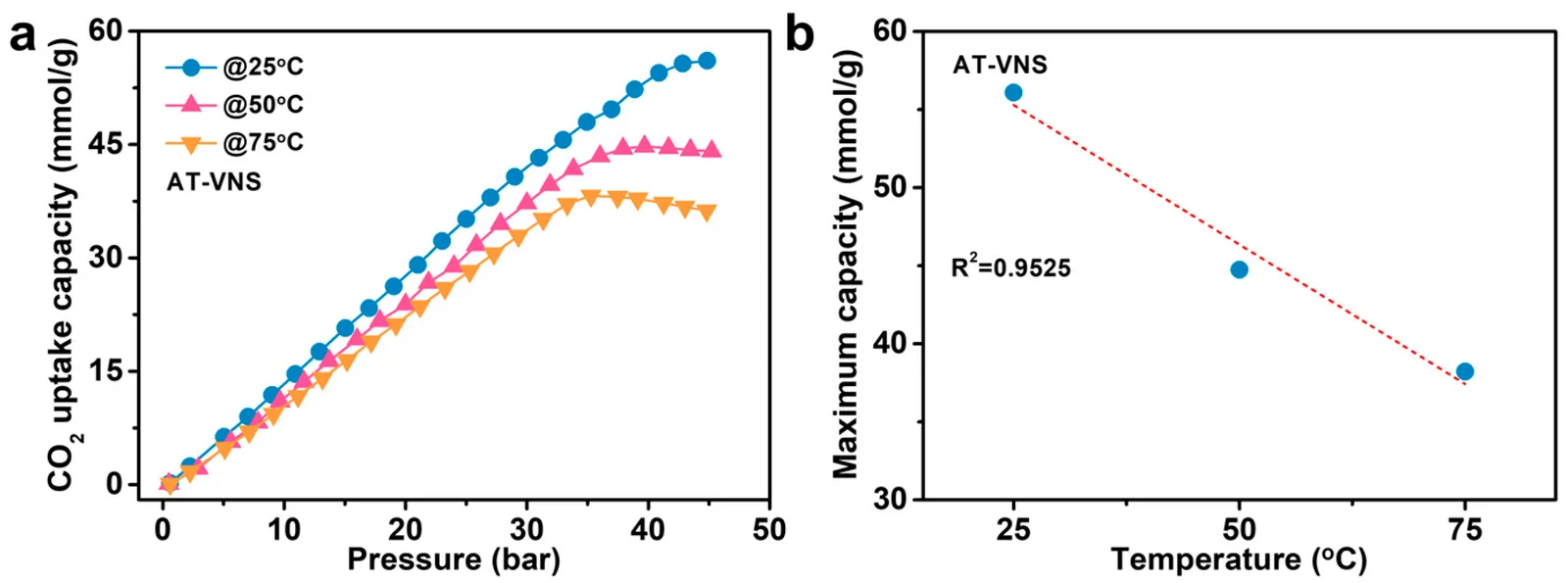
(**a**) CO_2_ adsorption isotherms of AT-VNS at different temperatures, and (**b**) the evolution of the maximum uptake capacity of AT-VNS with temperature.

The CO_2_ adsorption performance of AT-VNS at different temperatures was investigated; the results are shown in Figure 5. The uptake capacity exhibited an approximately linear increase with pressure across the range of 0–35 bar, indicating that AT-VNS provides sufficient adsorption sites for CO_2_ at low and medium pressures (Figure 5a). The maximum adsorption capacities at 25 °C, 50 °C, and 75 °C were 56.09 mmol/g, 44.74 mmol/g, and 38.22 mmol/g, respectively. Given that the physical adsorption of CO_2_ on AT-VNS is an exothermic process, an increase in temperature raises the internal kinetic energy of the gas molecules, thereby causing a slight loss in adsorption performance. A clear negative linear correlation was observed within the tested temperature range (Figure 5b). Even at the highest temperature (75 °C), AT-VNS still exhibited a high capture capacity of 38.22 mmol/g, demonstrating exceptional potential for high-temperature adsorption.

**Figure 6:**
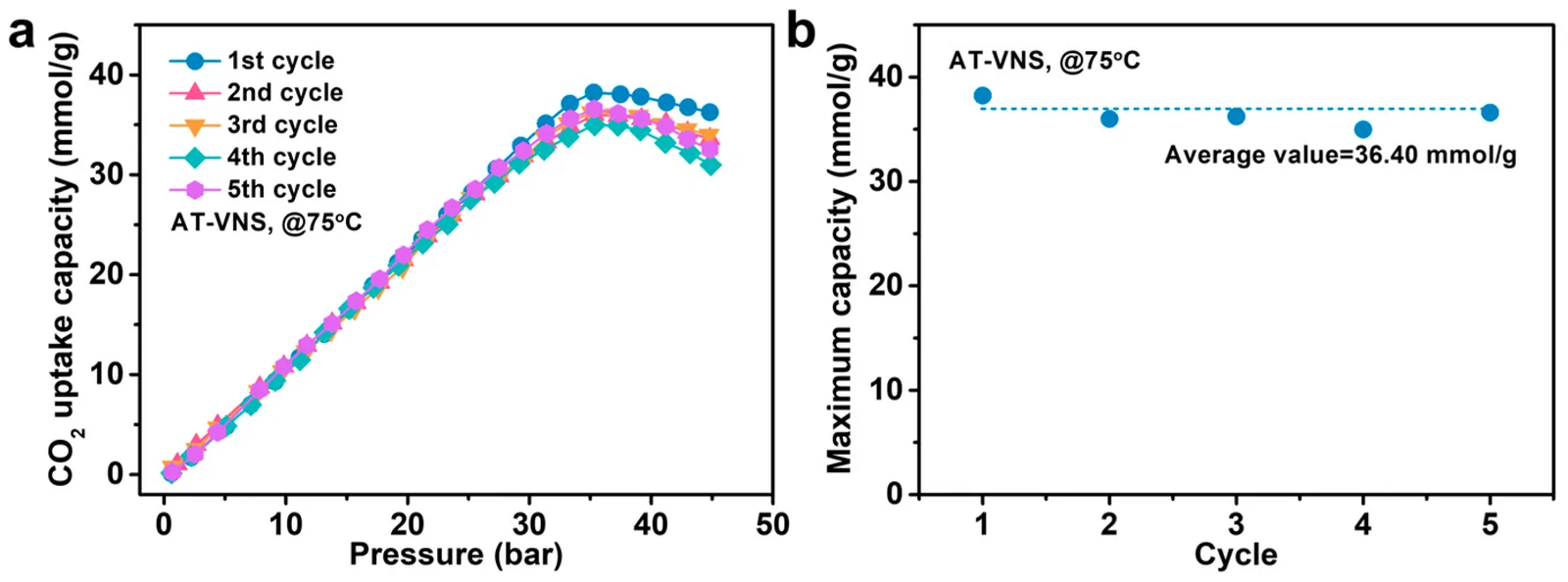
(**a**) CO_2_ adsorption isotherms and (**b**) maximum uptake capacity of AT-VNS at 75 °C over five cycles.

The cycle stability of CO_2_ adsorbents is crucial to their practical application. To further evaluate the cycle stability of AT-VNS, five adsorption–desorption cycles were performed at 75 °C, a temperature within the typical range for carbon capture from flue gas following industrial combustion. As shown in Figure 6a, similar CO_2_ adsorption isotherms were observed over five cycles. The excellent cycling performance is attributed to the stability of the AT-VNS pore structure and the rapid diffusion pathways it provides for gas molecules. The corresponding maximum uptake capacity was achieved at 35 bar and remained around 36.40 mmol/g (Figure 6b). Compared with the first cycle, the retention rate of the maximum uptake capacity in the fifth cycle reached as high as 95.2%. The slight loss in capture capacity can be attributed to the partial contraction or collapse of the interlayer pores during the adsorption–desorption cycles. The integration of high uptake capacity and excellent cyclic stability makes the AT-VNS promising as an efficient adsorbent for CO_2_ capture.

## 4. Conclusions

In summary, clay-based 2D porous nanosheets with a high specific surface area and a hierarchical porous structure were prepared using a strategy combining liquid-phase exfoliation with acid etching. We investigated the CO_2_ adsorption behavior of the clay-based porous nanosheets over a wide temperature range (25–75 °C). The clay-based porous nanosheets exhibited a high CO_2_ uptake capacity of 56.09 mmol/g at 25 °C and 45 bar, representing a 7.9-fold increase compared with the raw material. The microscopic mechanism underlying the enhanced CO_2_ capture performance of the porous nanosheets was elucidated. The study found that after undergoing five adsorption–desorption cycles at 75 °C, the CO_2_ uptake capacity of the porous nanosheets remained at approximately 36.40 mmol/g. The as-made porous nanosheets show great promise for CO_2_ capture due to the high uptake capacity and excellent cycling stability. Our research findings contribute to the rational design of highly efficient adsorbents, thereby promoting the development of CO_2_ capture and storage technologies.

## Figures and Tables

**Table 1:** Comparison of the CO_2_ uptake capacity of this work with previously reported results.

Solid Adsorbents	Adsorption Conditions	CO_2_ Uptake Capacity (mmol/g)	Year & Ref
AT-VNS	5 bar, 25 °C	6.32	This work
	25 bar, 25 °C	35.15	
	45 bar, 25 °C	56.09	
Biomass-derived porous carbon	10 bar, 0 °C	20.3	2022 [35]
Activated carbon	20 bar, 25 °C	23.2	2026 [36]
Zeolite template carbon	15 bar, 25 °C	17.38	2023 [37]
Metal–organic framework	5 bar, 25 °C	4.54	2024 [38]
Porous boron nitride	10 bar, 5 °C	4.95	2025 [39]

## Data Availability

The data presented in this study are available on request from the corresponding author.
